# The expression of LRRN4 was correlated with the progression and prognosis of colon adenocarcinoma (COAD) patients

**DOI:** 10.1590/1678-4685-GMB-2021-0138

**Published:** 2021-12-15

**Authors:** Yuxian Zhang, Jianlan Xie, Diangang Liu, Shengtao Zhu, Shutian Zhang

**Affiliations:** 1 Beijing Key Laboratory for Precancerous Lesion of Digestive Disease, Beijing Digestive Disease Center, National Clinical Research Center for Digestive Disease, Capital Medical University, Beijing Friendship Hospital, Department of Gastroenterology, Beijing, P. R. China.; 2 Capital Medical University, Beijing Friendship Hospital, Department of Pathology, Beijing, P.R. China.; 3 Xuanwu Hospital, Capital Medical University, Department of General Surgery, Beijing, P. R. China.

**Keywords:** Colon adenocarcinoma, LRRN4, prognosis, biomarker

## Abstract

Our present study aims to investigate the value of LRRN4 in the progression and prognosis of COAD patients. All COAD and adjacent sample data was downloaded from TCGA database. Survival analysis was performed according to Kaplan-Meier method. The real-time quantitative PCR and immunohistochemistry analysis were conducted for validation in cell lines and tissues. The GSEA was conducted to find functional KEGG pathways. Multivariate Cox regression proportional hazard mode was used to determine whether LRRN4 expression was an independent prognostic factor. The LRRN4 expression in COAD samples were significantly higher than that in adjacent samples, which was consistent with our experiments in cell lines and tissues. Along with the increase of TNM Stage, LRRN4 expression had an increasing tendency. The COAD patients with high LRRN4 expression showed undesirable prognoses. Additionally, the TGF-β signaling pathway, WNT signaling pathway and other 25 pathways were significantly activated in the high LRRN4 expression group. In conclusion, high LRRN4 expression was closely related to the onset of COAD and it was a poor prognostic factor for COAD patients.

## Introduction

Colon adenocarcinoma (COAD), is the dominant type of colon cancer (Mutch, 2007), which is one of the most common gastrointestinal tumors around the world (Arnold *et al*., 2017). As lots of factors affect the development of COAD, for example, alcohol, obesity and so on, the prevalence of COAD is high and growing annually (Islami *et al*., 2018; Siegel *et al*., 2019). In addition, COAD is a highly invasive adenocarcinoma and has great heterogeneity (Kalyan *et al*., 2018; Sun *et al*., 2016;Yang *et al*., 2019), which brings great challenges to the early diagnosis and treatment of COAD patients. The early detection of colon cancer would help improve overall survival after comparing the patients diagnosed at different stages (Lee *et al*., 2020). The progression of COAD is usually a multi‐stage process (Mutch, 2007), which reminds both COAD patients and researchers that it is important to take actions to prevent and diagnose early. Thus, there is no doubt that early detection and diagnosis is quite necessary for patients. With the development of medical technology, possible biomarkers identification is a promising tool for COAD diagnosis and prognosis. Accordingly, our team had spared no efforts to look for the reliable biomarkers in colon cancer before, and we have reported several miRNAs and genes (Li *et al*., 2020; Min *et al*., 2019). Besides, some other previous studies have demonstrated that aberrant gene expressions, such as TROP2 (Zhao and Zhang, 2018), HIF‐1 (Sun *et al*., 2020), played a crucial role in the onset of COAD. However, more specific biomarkers of COAD are still urgently needed.

LRRN4 (leucine rich repeat neuronal 4), a newly identified member of leucine rich repeat neuronal protein family (NLRR), has been reported to be expressed in various tissues. At present, LRRN4 has been investigated mainly in the central nervous system (CNS) (Bando *et al*., 2005) and the peripheral nervous system (PNS) (Bando *et al*., 2012). A recent study revealed that the aberrantly low expression of LRRN4 was closely associated with the dilated cardiomyopathy (Li *et al*., 2017), which reminded us that aberrant LRRN4 expression might also play a role in other diseases. To the best of our knowledge, LRRN4 has not been studied in cancers, but several other members of NLRR have been reported in some cancers. For example, the expression of LRRN1 was upregulated in gastric cancer tissues and LRRN1 was related to the poor prognosis (Liu *et al*., 2019). Not only that, NLRR1 was reported to be an extracellular negative regulator of ALK signaling in neuroblastoma (Satoh *et al*., 2016). In another study, NLRR1, NLRR3 and NLRR5 were found to have different biological functions among the neuroblastoma subsets (Hamano *et al*., 2004). However, LRRN4 has not been well explored in COAD yet. Collectively, the role of LRRN4 in COAD progression and prognosis should be well explored in the future for getting more information of LRRN4 and COAD.

Herein, based on the COAD-related data downloaded from The Cancer Genome Atlas (TCGA) database, we proposed to investigate the value of LRRN4 in the progression and prognosis of COAD patients, for convenience to supply alternative biomarkers for COAD patients and to understand the mechanism behind COAD.

## Material and Methods

### TCGA data

All data were downloaded from The Cancer Genome Atlas (TCGA, https://tcga-data.nci.nih.gov/tcga/) database. The mRNA expression data and corresponding clinical information of 456 COAD patients were obtained, including 456 COAD cancer tissues and 41 paired adjacent tissues. Among which, 433 COAD samples with complete survival information were used for further analysis. According to the median of LRRN4 expression, all cancer samples were divided into high and low LRRN4 expression COAD specimens. Detailed clinical information of 433 patients was showed in Table 1. Besides, 179 rectum adenocarcinoma (READ) patients’ mRNA and clinical information data was also downloaded.

**Table 1 -:** Clinical characteristics of patients with colon adenocarcinoma based on the TCGA database.

Clinical characteristics		Total (n=433)	Percent(%)
Age	Median[Min,Max]	68[34,90]	
Gender	Male	233	53.82
	Female	200	46.18
TNM Stage	Stage I	73	16.86
	Stage II	165	38.12
	Stage III	123	28.41
	Stage IV	61	14.09
	Unknown	11	2.54
T Stage	T1	11	2.54
	T2	75	17.32
	T3	296	68.36
	T4	50	11.55
	Tis	1	0.23
N Stage	N0	254	58.66
	N1	102	23.56
	N2	77	17.78
M Stage	M0	320	73.90
	M1	61	14.09
	MX	45	10.39
	Unknown	7	1.62
Race	American indian or alaska native	1	0.23
	Asian	11	2.54
	Black or african american	56	12.93
	white	209	48.27
	Unknown	156	36.03
History of colon polyps	NO	238	54.97
	YES	128	29.56
	Unknown	67	15.47
Disease type	Adenomas and Adenocarcinomas	369	85.22
	Epithelial Neoplasms	3	0.69
	Mucinous and Serous Neoplasms	61	14.09
BMI	Median[Min,Max]	27.13[14.72,271.86]	
Vital status	Alive	338	78.06
	Dead	95	21.94

### Colon adenocarcinoma patients

COAD tissues and adjacent tissues were collected from 15 patients diagnosed by two pathologists in Beijing Friendship Hospital from May 2020 to Dec 2020. The clinical validations were approved by the Ethics Committee of Beijing Friendship Hospital according to the Declaration of Helsinki (ethic code: 2017-P2-013-03), and informed consents were signed by all patients. The clinical information of patients were shown in the Table S1.

### Survival analysis

Base on the Kaplan-Meier method, the overall survival (OS) probability of high and low LRRN4 expression group was estimated using survival package and survminer package (https://CRAN.R-project.org/package=survminer) in R language. The OS probability difference between different groups were determined by log-rank.

### Cell culture

Human colonic mucosa cell line NCM460 and colon cancer cell line SW480 were purchased from Chinese Academy of Sciences (Shanghai, China), and verified by the Single Tandem Repeat (STR) profiling method. Both cell lines were cultured in complete DMEM medium, consisting of DMEM (Gibco, MA, USA) and 10% fetal bovine serum (Gibco, MA, USA). The cells were maintained in 37 ℃ saturated humidified environment with 5% CO_2_.

### RNA extraction and the real-time quantitative PCR

For total RNA extraction and quantification, Trizol reagent (Cat# 15596-026, Invitrogen, Grand Island, CA, USA) and Nanodrop lite (Thermo, USA) were used. For reverse transcription, complementary DNA (cDNA) was obtained by TIANScript RT kit (Cat# KR104-02, TIANGEN, Beijing, China). The real-time quantitative PCR amplification was performed by SuperRealPreMix Plus kit (Cat# FP205, TIANGEN, Beijing, China), and GAPDH was an internal control. The primers of LRRN4 and GAPDH are listed in Table 2. All samples were analyzed by the LightCycler480 (Roche, USA) in biological triplicates for 40 cycles. The data of real-time quantitative PCR analysis was calculated by the 2^-△△Ct^ method.

**Table 2 -:** Primer sequences.

Genes	Forward Primer (5’-3’)	Reverse Primer (5’-3’)	Product length (bp)	Tm(℃) (◦C)
LRRN4	CGTGGGACCGCAGCATAAGC	CCTTCCTCCTCCTCACTGTAGTCG	133	60
GAPDH	GCAAATTCCATGGCACCGTC	AGCATCGCCCCACTTGATTT	110	60

### Immunohistochemistry (IHC) analysis

IHC validation was conducted as described (Zeng *et al*., 2017). Antibody against LRRN4 was purchased from Abcam (ab133372, 1:100, Shanghai, China), and Goat anti-Rabbit IgG (H+L) -HRP were obtained from Bioworld (Cat#BS13278, 1:1000, Nanjing, China).

### Gene set enrichment analysis (GSEA)

Based on the gene set c2.cp.kegg.v7.0.symbols from Molecular Signatures Database (MSigDB), GSEA was performed using software GSEA (version: #4.0). The KEGG pathways with P value <0.05 were considered as significant enrichment.

### Statistical analysis

The LRRN4 expression in cancer samples, adjacent to normal samples and samples with various clinicopathological characteristics were compared using Wilcoxon rank-sum test. The influence of LRRN4 expression and different clinicopathological characteristics (Age, Sex, Stage, etc.) on OS was determined by the Multivariate cox regression proportional hazard mode. The difference was considered to be statistically significant if P value ≤0.05. R software (version 3.5.2.) was used for all statistical analysis.

### Ethics

All procedures followed were in accordance with the Ethics Committee of Beijing Friendship Hospital according to the Declaration of Helsinki (ethic code: 2017-P2-013-03).

### Data availability

The datasets used and analysed in the present research were downloaded from The Cancer Genome Atlas (TCGA, https://tcga-data.nci.nih.gov/tcga/) database.

## Results

### High LRRN4 expression was closely associated with the occurrence of COAD

**Figure 1 -:**
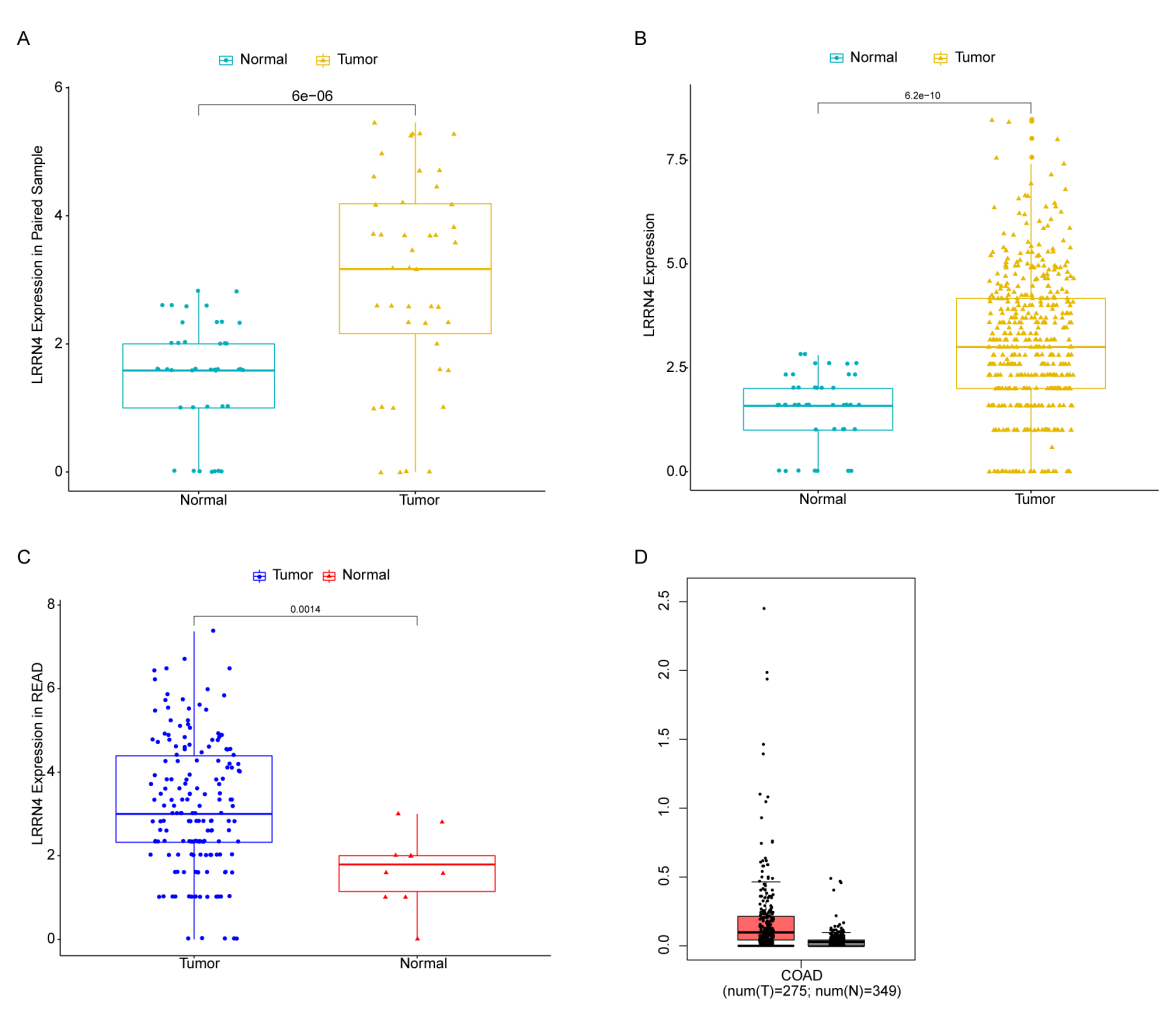
The expression levels of LRRN4 in COAD and adjacent normal samples. (A) Box plot of LRRN4 expression in paired COAD and adjacent normal samples. (B) Box plot of LRRN4 expression in all COAD samples and adjacent normal samples. (C) LRRN4 expression in READ samples and adjacent samples. (D) In GEPIA database, LRRN4 expression in COAD samples and normal samples.

In order to explore the relationship between LRRN4 expression and the onset of COAD, LRRN4 expression in COAD samples was compared with that in adjacent samples. The results showed that the expression of LRRN4 in COAD samples (N = 41) was significantly higher than that in paired adjacent samples (P = 6e-06) (Figure 1A). In addition, the expression of LRRN4 in all COAD samples (N = 433) was also significantly higher than that in adjacent samples (P=6.2e-10) (Figure 1B). Moreover, the LRRN4 expression was also significantly higher in READ samples when compared with adjacent samples (Figure 1C). The same tendency was also observed in GEPIA database (http://gepia.cancer-pku.cn/) (Figure 1D). These results suggested that high LRRN4 expression was closely associated with the onset of COAD.

### The association between LRRN4 expression and clinicopathological characteristics

**Figure 2 -:**
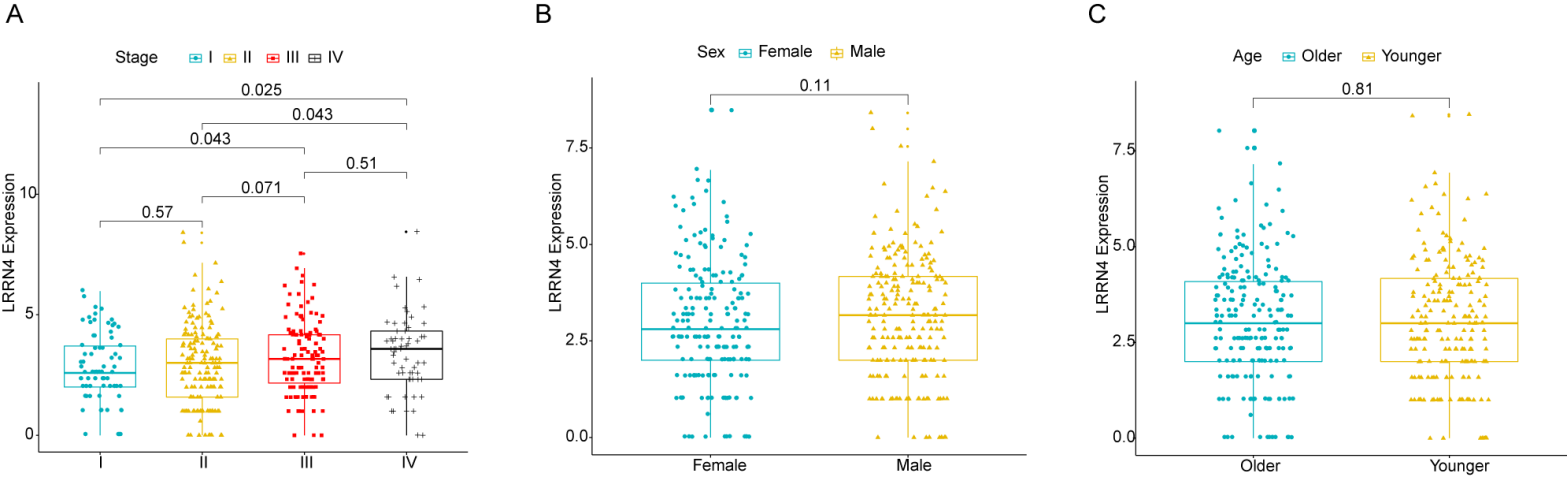
The association between LRRN4 expression and clinicopathological characteristics**.** (A) The box plot of LRRN4 expression levels in different TNM stages. (B) The box plot of LRRN4 expression levels in different genders. (C) The box plot of LRRN4 expression levels in different ages.

To investigate the association between LRRN4 expression and clinicopathological characteristics, 433 COAD samples were analyzed using Wilcoxon rank-sum test. The results showed that along with the increase of TNM Stage, LRRN4 expression had a growing tendency. There was a significantly statistical difference in Stage I vs. Stage III, Stage II vs. Stage IV and Stage I vs. Stage IV (P value < 0.05, Figure 2A). LRRN4 expression was not significantly related to Sex and Age (P value > 0.05, Figure 2B and 2C).

### High LRRN4 expression COAD patients showed poor prognosis

**Figure 3 -:**
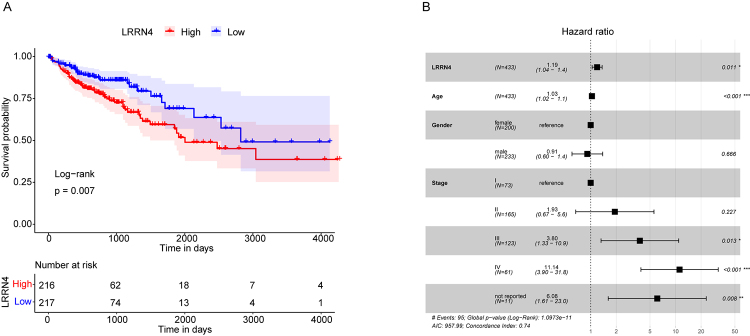
The prognosis of COAD patients in high LRRN4 expression group was poor. (A) Kaplan Meier survival curve of patients with high and low LRRN4 expression. P value was determined by log-rank test. X-axis: time; y-axis: survival probability; color: various groups. (B) Multivariate cox regression analysis forest plot. Compared with reference samples, samples with Hazard ratio greater than 1 had a higher risk of death, and samples with a Hazard ratio less than 1 had a lower risk of death.

To explore the effect of LRRN4 expression on the prognosis of COAD patients, a survival analysis was conducted on high and low LRRN4 expression patients in the TCGA database. Compared with low LRRN4 expression patients, highly LRRN4 expressing patients had a poorer OS (p = 0.007, HR=0.57, 95%CI: 0.38-0.85) (Figure 3A). 

To confirm whether LRRN4 expression was an independent prognostic indicator, a multivariate Cox regression analysis, including Age, Sex, Stage and LRRN4, was conducted. The results showed that LRRN4 expression was still significantly correlated with the OS of COAD patients. High LRRN4 expression samples had a higher risk of death, which indicated that high LRRN4 expression was a poor prognostic factor (HR=1.19, 95%CI: 1.04-1.4, P = 0.011) (Figure 3B).

### LRRN4 was upregulated in colon cancer cell lines and clinical COAD tissues

**Figure 4 -:**
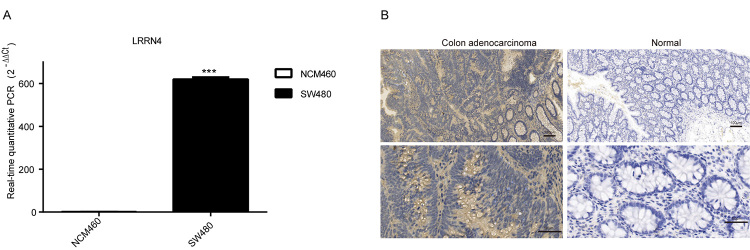
LRRN4 was upregulated in colon cancer cell lines and COAD patients. (A) the relative mRNA expression level of LRRN4 in colon cancer cell lines. *** P < 0.001. (B) representative IHC images of LRRN4 expression in COAD patients.

To validate our results obtained from the public dataset, experiments were conducted for detecting the LRRN4 expression level in colon cancer cell lines and clinical COAD tissues. The results suggested that the relative mRNA expression of LRRN4 (Figure 4A, P value <0.001) was high in colon cancer cell lines, which was consistent with the analysis of IHC. As shown in Figure 4B, pathologically incomplete intestinal gland was in tumor region, and IHC analysis indicated that LRRN4 was upregulated in COAD tissues, compared to the corresponding normal tissues. Thus, our findings showed that the upregulation of LRRN4 might be involved in the progression of COAD.

### Signaling pathways related to LRRN4 expression based on GSEA

**Figure 5 -:**
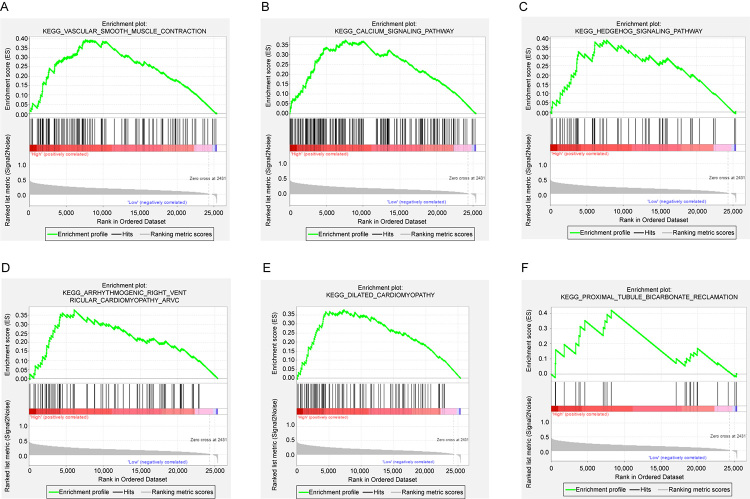
The GSEA results of LRRN4 expression.

In TCGA database, GSEA enrichment analysis was used to identify the signaling pathways significantly activated in high LRRN4 expression samples relative to low LRRN4 expression samples. P value <0.05 was taken as criteria to screen significantly enriched KEGG pathways. The results showed that VASCULAR_SMOOTH_MUSCLE_CONTRACTION, CALCIUM_SIGNALING_PATHWAY, HEDGEHOG_SIGNALING_PATHWAY, ARRHYTHMOGENIC_RIGHT_VENTRICULAR_CARDIOMYOPATHY_ARVC, DILATED_CARDIOMYOPATHY, PROXIMAL_TUBULE_BICARBONATE_RECLAMATION and other 21 pathways were significantly activated in high LRRN4 expression samples relative to low LRRN4 expression samples (Table S2). The top six most significantly enriched pathways are displayed in Figure 5A-5F.

## Discussion

In the present study, we have firstly investigated the role of LRRN4 in the development and prognosis of COAD patients, utilizing a series of bioinformatic analyses and experimental validation. High LRRN4 expression was closely related to the onset of COAD. Not only that, highly LRRN4 expressed COAD patients showed relatively undesirable prognosis, compared with low LRRN4 expression COAD patients.

Firstly, the association between LRRN4 expression with the onset of COAD was investigated. According to the analyses of COAD related data, LRRN4 expression in COAD tissues was significantly higher than that in both paired adjacent samples and all adjacent samples, which has also been successfully validated from mRNA and protein level. Our findings showed that high LRRN4 expression was closely associated with the occurrence of COAD. As far as we know, this is the first time LRRN4 has been studied in COAD. LRRN4, as a member of LRRN family, was mainly reported in regulation of cardiac diseases (Brody and Lee, 2016; Moc *et al*., 2015), central nervous system (CNS) (Bando *et al*., 2005) and the peripheral nervous system (PNS) (Bando *et al*., 2012). For instance, a recent study reported that the aberrantly low expression of LRRN4 was closely associated with the dilated cardiomyopathy (Li *et al*., 2017), which reminded us that aberrant LRRN4 expression might also play a role in other diseases. In our study, aberrantly high LRRN4 expression was associated with the onset of COAD, which seemed to play a role in a similar way. Additionally, some other members of the LRRN family were reported in some cancer studies. LRRN1 was involved in gastric cancer (Liu *et al*., 2019) and neuroblastoma (Hamano *et al*., 2004; Satoh *et al*., 2016). Moreover, it has been documented that regulatory signals of the enteric innervation might be related to the pathogenesis of colorectal cancer (Sitohy and El-Salhy, 2002). Since the role of LRRN4 in nervous system, we suspected that LRRN4 might influence the onset of COAD in an indirect way, which still needs lots of further researches in the future.

Additionally, the correlation between LRRN4 expression and various clinicopathological characteristics was investigated in all COAD samples. We found that along with the increase of TNM Stage, LRRN4 expression had a growing tendency. But there was no significant association between LRRN4 expression and sex, age. TNM staging was usually evaluated after the operation and it was a crucial prognostic factor (Mokhtari and Zakerzade, 2017), which indicated that TNM stage was an important aspect for COAD prognosis. Subsequently, via survival analyses, highly LRRN4 expressing COAD patients were found to have a poorer OS, compared with low LRRN4 expression patients. Through the multivariate Cox regression analysis, LRRN4 expression was still an independent prognostic indicator for the prognosis of COAD. Collectively, high LRRN4 expression was a poor prognostic factor for COAD. 

Based on the GSEA enrichment analysis, we have identified 27 signaling pathways that were significantly activated in high LRRN4 expression samples relative to low LRRN4 expression samples. Among which, several pathways caught our attention, such as TGF-β signaling pathway, gap junction, WNT signaling pathway and so on. TGF-β pathway was previously evidenced to be involved in primary tumor progression and in promoting metastasis in many human cancers including colorectal cancer (Akbari *et al*., 2014; Akhurst, 2017; Korkut *et al*., 2018). In this study, the TGF-β pathway was significantly activated in the high LRRN4 expression group, which was consistent with former studies. Regarding gap junction, it has been reported that along with the progression of colorectal neoplasia, some functional gap junctions were gradually lost (Kanczuga-Koda *et al*., 2010), while the mechanism behind the gap junction pathway in COAD were still not clear. A recent study has revealed that the activity of Wnt/β-catenin signaling could be regulated by certain genes or microRNAs, which would further regulate the progression of colorectal cancer, including COAD (Han *et al*., 2011; Shao *et al*., 2019). Consequently, there is a complicated regulation network influenced by the LRRN4 expression, which was still not clear and deserves further efforts.

## Conclusions

We have firstly investigated the role of LRRN4 in COAD based on the bioinformatic analyses of COAD related data in TCGA database and further experimental validation. We found that high LRRN4 expression was probably related to the onset of COAD and highly LRRN4 expresseing COAD patients had undesirable OS. LRRN4 might be a promising prognostic biomarker in COAD patients, which deserves further exploration.

## Supplementary material

The following online material is available for this article:

Table S1 - The clinical information of patients.

Table S2 - Result of GSEA.
