# Supplementary material for: The expression of LRRN4 was correlated with the progression and prognosis of colon adenocarcinoma (COAD) patients
Source: Genet Mol Biol. 2021 Dec 15;45(1):e20210138. doi: 10.1590/1678-4685-GMB-2021-0138 (PMC8679243; doi:10.1590/1678-4685-GMB-2021-0138)
Supplement: Table S1 [file 1415-4757-GMB-45-1-e20210138-s1.pdf]

Genetics and Molecular Biology
https://doi.org/10.1590/1678-4685-GMB-2021-0138

Supplementary Material to "The expression of LRRN4 was correlated with the progression and prognosis of colon adenocarcinoma (COAD) patients"

Table S1. The clinical information of patients.

No.	TNM Stage	Age	Gender	Size (cm)
1	T2N0M0	46	Female	3.5*2.5*0.7
2	T3N1bM0	57	Male	4*3.3*1.5
3	T3N0M0	63	Male	2.3*2.2*0.4
4	T3N1bM0	69	Male	2.7*2.2*1
5	T3N1aM0	64	Female	3.5*2.5*0.5
6	T3N0M0	60	Female	1.8*1.5*0.4
7	T3aN0M0	67	Female	4*3.2*1.2
8	T3N0M0	63	Male	6*4*2
9	T3N0M0	67	Female	2*1*0.8
10	T3N1bM0	70	Male	4.5*2*0.5
11	T3N1aM0	63	Female	4*4*1.5
12	T1b-sm3N0M0	69	Female	2*1.5*0.8
13	T4N1aM0	70	Male	5.5*2.5*0.5
14	T3N0M0	63	Male	2.7*2.7*1
15	T4aN1bM0	60	Male	3.5*3.5*2
