# Supplementary material for: The expression of LRRN4 was correlated with the progression and prognosis of colon adenocarcinoma (COAD) patients
Source: Genet Mol Biol. 2021 Dec 15;45(1):e20210138. doi: 10.1590/1678-4685-GMB-2021-0138 (PMC8679243; doi:10.1590/1678-4685-GMB-2021-0138)
Supplement: Table S2 [file 1415-4757-GMB-45-1-e20210138-s2.pdf]

Genetics and Molecular Biology
https://doi.org/10.1590/1678-4685-GMB-2021-0138

Supplementary Material to “The expression of LRRN4 was correlated with the progression and prognosis of colon adenocarcinoma (COAD) patients”

Table S2. Result of GSEA

NAME	ES	Pvalue
KEGG_VASCULAR_SMOOTH_MUSCLE_CONTRACTION	0.39410788	0.013653846
KEGG_CALCIUM_SIGNALING_PATHWAY	0.3718467	0.025626203
KEGG_HEDGEHOG_SIGNALING_PATHWAY	0.38766333	0.02237354
KEGG_ARRHYTHMOGENIC_RIGHT_VENTRICULAR_CARDIOMYOPATHY_ARVC	0.37904704	0.027920792
KEGG_DILATED_CARDIOMYOPATHY	0.37824	0.02963671
KEGG_PROXIMAL_TUBULE_BICARBONATE_RECLAMATION	0.41996977	0.028875968
KEGG_INOSITOL_PHOSPHATE_METABOLISM	0.32740155	0.03015564
KEGG_HYPERTROPHIC_CARDIOMYOPATHY_HCM	0.36612102	0.031287128
KEGG_ECM_RECEPTOR_INTERACTION	0.37682167	0.038582677
KEGG_GLYCOSAMINOGLYCAN_BIOSYNTHESIS_HEPARAN_SULFATE	0.36476317	0.032954547
KEGG_TGF_BETA_SIGNALING_PATHWAY	0.3111419	0.034416825
KEGG_BASAL_CELL_CARCINOMA	0.361404	0.034714004
KEGG_PHOSPHATIDYLINOSITOL_SIGNALING_SYSTEM	0.30180252	0.037058824
KEGG_GAP_JUNCTION	0.3068206	0.036023623
KEGG_WNT_SIGNALING_PATHWAY	0.26009095	0.037401575
KEGG_FOCAL_ADHESION	0.2860488	0.044186047
KEGG_MELANOMA	0.30028185	0.04481409
KEGG_REGULATION_OF_ACTIN_CYTOSKELETON	0.25644544	0.044357976
KEGG_GLYCOSAMINOGLYCAN_BIOSYNTHESIS_CHONDROITIN_SULFATE	0.38409877	0.0464
KEGG_MELANOGENESIS	0.28089413	0.041472867
KEGG_CARDIAC_MUSCLE_CONTRACTION	0.29329556	0.043713734
KEGG_LINOLEIC_ACID_METABOLISM	0.38840503	0.044038463

KEGG_NITROGEN_METABOLISM	0.36796463	0.043575418
KEGG_NOTCH_SIGNALING_PATHWAY	0.2816431	0.047490346
KEGG_DORSO_VENTRAL_AXIS_FORMATION	0.30393672	0.047900763
KEGG_BASAL_TRANSCRIPTION_FACTORS	0.31394172	0.049510762
KEGG_LONG_TERM_DEPRESSION	0.28804755	0.048183557
